# Editorial Comment on Frequency of tethered cord syndrome in pediatric patients with refractory daytime urinary incontinence

**DOI:** 10.1111/iju.15268

**Published:** 2023-08-18

**Authors:** Hiroshi Shimura

**Affiliations:** ^1^ Department of Urology University of Yamanashi Graduate School of Medical Sciences Yamanashi Japan

The author described the prevalence of tethered cord syndrome (TCS) in patients with refractory daytime urinary incontinence (DUI).^1^ It should be kept in mind that in pediatric patients with unexplained lower urinary tract dysfunction (LUTD) at initial evaluation, neurological disorders such as TCS may be latent. However, until now, the prevalence of TCS in such patients has been unclear, and invasive tests such as pressure flow study (PFS) have been performed without any consideration. The frequency in this study, at 9%, appears to be somewhat higher than previously reported.^2^, ^3^ The data in this study were collected from a relatively large number of patients who underwent MRI and PFS, and the results are very valuable.

Interestingly, TCS cases were found in cases where uroflowmetry showed no apparent abnormality.^1^ In other words, it seems difficult to detect TCS cases by screening tests. This study is significant as an evidence for explaining to patients and the parents the need for testing and the usefulness of the information obtained from examinations of MRI and PFS. The author suggests that MRI as a screening test be performed prior to PFS, especially in children, as it requires careful consideration prior to invasive testing.

However, in my personal opinion, PFS is a significant examination even it is invasive, because PFS results can also lead to the possibility of diseases other than TCS. Considering the possibility of occult TCS (OTCS) not confirmed by MRI, PFS is still important. In the case of refractory LUTD, I could not recommend avoiding PFS. In any case, care must be taken not to miss TCS, which is expected to benefit from surgical therapy so that appropriate testing can be performed at the appropriate time. While the results of this study revealed the prevalence of TCS in children, I personally hope that further research will also reveal the hidden prevalence of TCS in adults.

## AUTHOR CONTRIBUTIONS

**Hiroshi Shimura:** Writing—original draft; Writing—review & editing.

## CONFLICT OF INTEREST STATEMENT

None declared.
